# Managing wildlife populations with uncertainty: cormorants *Phalacrocorax carbo*

**DOI:** 10.1111/j.1365-2664.2008.01380.x

**Published:** 2008-12

**Authors:** G C Smith, D Parrott, P A Robertson

**Affiliations:** Central Science LaboratorySand Hutton, York Y041 1LZ, UK

**Keywords:** density dependence, model structure, Monte Carlo model, population, population growth, population model, population index

## Abstract

1. Managing wildlife populations for conservation, control or harvesting involves uncertainty. Nevertheless, decisions need to be made based on the available evidence. The two main sources of uncertainty in population modelling are parameter estimates and structural uncertainty. Structural uncertainty in models is not included as often as parameter uncertainty.
2. We present an approach where parameter and structural uncertainty (strength of density dependence) is included within a model, using the over-wintering English population of cormorants *Phalacrocorax carbo* L. Because of the damage caused to inland fishery interests by cormorants, there was a change in UK government policy in autumn 2004, increasing the numbers of birds that can be shot under licence.
3. A stochastic Monte Carlo annual population model was produced to examine the effect of changes to the numbers of birds shot each year. Indices of annual population size were converted to population estimates and used to determine annual growth rates and strength of density dependence.
4. There is strong evidence for density dependence in the data, which suggests the population is currently slightly above carrying capacity, with a mean growth rate of 4–6% per annum. The 1300 birds shot under licence in 2004/05 represent about 4·5% of the English population, and if this level of culling continues, the population would be expected to decline by 9% by 2007, compared to the long-term average. The a priori preferred model, which included all uncertainty, gave predictions for 2004/05 and 2005/06 in close agreement with field data.
5. The model was used to produce short-term population projections, with the understanding that Adaptive Resource Management (ARM) will be adopted to iteratively update the parameters and model each year, feeding back into the numbers of available licences.
6. *Synthesis and applications*. We recommend the approach used in this study of including parameter and structural uncertainty within a single model, where possible, with the proportion of iterations that utilize a particular structure dependent on the weight of evidence for that structure. This will produce results with wider confidence intervals, but ensures that the evidence for any particular model is not over-interpreted.

## Introduction

When managing wildlife populations, although inherent uncertainty exists in our knowledge, decisions need to be made based on the available evidence, even if that decision is to delay management until further data are available. It may be necessary, therefore, to make a decision using data that may contain bias or error; thus, wherever possible, this should be accounted for. Nonetheless, we can be forced to make decisions using data that are less than perfect. This is critically true for rare and endangered species, but also true for many species that are managed due to their status as an exploited or pest species. For endangered species, the most commonly used approach is Population Viability Analysis or PVA (Lindenmayer *et al*. 1993; Akçakaya & Burgman 1995; Brook *et al*. 2000), which predicts the risk of population decline below a set threshold. PVAs have also been used to assess the success of reintroductions (Leaper *et al*. 1999), but not commonly on the management of pests. Pest and disease control is most often modelled with ordinary or partial differential equations (Anderson & May 1979), or individual-based (IB) models (DeAngelis & Gross 1992;Shirley *et al*. 2003).

There are two main sources of uncertainty in population models; (i) parameter uncertainty and (ii) structural uncertainty. Parameter uncertainty is often evaluated with sensitivity analysis, although there is no general acceptance of which of the many methods to use (Saltelli, Chan & Scott 2000). Structural uncertainty is less often evaluated (but see Todd *et al*. 2001; Brugnach 2005) and there are no accepted methods for dealing with it, despite the fact that models can be very sensitive to structural assumptions (Wood & Thomas 1999). Comparison between models, using different approaches, is uncommon (e.g. Topping *et al*. 2005).

Models are often produced based on a single approach and structure, although model structure could be treated as a separate hypothesis for both PVA (Reed *et al*. 2002) and IB (Smith *et al*. 1995) models, and the mathematical function describing density dependence may be critical to the output of such models (Henle, Sarre & Wiegand 2004). Historical population counts are often used to determine growth rates, and methods exist to estimate density-dependent and density-independent annual growth rates in the presence of sampling or observational error (see Freckleton *et al*. 2006).

One way of dealing with structural uncertainty is to build multiple models that include all realistic structures and base management decisions on the results, for example, the time to reach a quasi-extinction threshold for the eradication of an invasive species (Smith, Henderson & Robertson 2005). Another approach is to choose between multiple models, or to weight their output with an information-theoretic approach (Burnham & Anderson 2002). Here, we take the model of Smith *et al*. (2005) and apply the principle of multi-model inference within the structure of the model. We use the output to evaluate the viability of the great cormorant *Phalacrocorax carbo* L. under different levels of population culling.

In Europe, in recent decades there has been a marked increase in numbers of the two European subspecies of the great cormorant, the ‘Atlantic’ race *P. c. carbo* L. and the ‘Continental’ race *P. c. sinensis*Blumenbach (Debout, Rov & Sellars 1995; Lindell *et al*. 1995; Van Eerden & Gregersen 1995). The UK cormorant population is dominated by *P. c. carbo* (Cramp & Simmons 1977; Marion & Le Gentil 2006) and the conservation status of the cormorant in the European Union is favourable, with the population increasing and its threat status secure (BirdLife International 2004).

In the UK, an increase in the number of birds, range expansion, and changes in seasonal distribution have brought cormorants into conflict with inland fisheries. These conflicts generally occur during winter when numbers of cormorants inland are greatest. In England and Wales, in addition to increased numbers of birds wintering inland, the number of inland waters occupied by cormorants has also increased (Kershaw & Hughes 1997; Wernham *et al*. 1999).

Fisheries managers claim that increased predation by cormorants has a detrimental impact, and case studies have described predation levels that cause serious damage (Feltham *et al*. 1999). Similar concerns are widespread throughout Europe (Marquiss & Carss 1994; Carss 2002). A review of 25 European countries (Carss 2002) indicated that shooting adult cormorants in the non-breeding season represented the most common control method. Cormorants are fully protected under the European Community Directive on the Conservation of Wild Birds (EEC/79/409), except by derogation. In 14 countries, there are regulations that allow the culling of cormorants. In six countries (including the UK and Spain), licenses may be issued for the killing of a limited number of cormorants at specific sites to enhance scaring activities. In most countries, however, there is no assessment of the effects of shooting on the population (Frederiksen, Lebreton & Bregnballe 2001).

In Britain, the Wildlife and Countryside Act (1981) implements the European Community Directive on the Conservation of Wild Birds and allows the killing of cormorants under licence for certain purposes. Licences to kill cormorants are usually granted to prevent serious damage to fisheries. They permit the lethal shooting of a specified number of cormorants at specific sites, with applications assessed on a case-by-case basis. Until recently, the role of shooting to kill cormorants under licence was solely to reinforce non-lethal scaring methods. In autumn 2004, amendments to the cormorant licensing policy were introduced, and the Department for Environment, Food and Rural Affairs (Defra) now grants licences that allow the shooting of up to 2000 cormorants annually, with up to 3000 in the first few years (Defra 2004), both to reinforce the effects of non-lethal scaring measures and also to directly limit cormorant numbers at specific sites. With an increase in the number of cormorants that may be killed each year, it is important to assess the effects on the population.

## Methods

Suitable models can be produced using annual population count data, or demographic data (brood size, probability of breeding and mortality rates). Models based on population counts are simpler, require less information (one algorithm for density dependence rather than three) and the available data are more suitable. Although data exist on cormorant breeding (Newson *et al*. 2005; Bregnballe 2006) and survival (Wernham *et al*. 1999; Henaux, Bregnballe & Lebreton 2007), more data are available from population indices. Therefore, an annual population model was utilized.

When constructing a population model, it is important to know whether the population is density dependent or density independent, as this may result in marked differences in the forward projection of that population following management. A stochastic model can calculate the probability of any given outcome occurring. The approach taken here follows Smith *et al*. (2005) except that a single model was constructed which encompassed the uncertainty of density dependence and population size. The model was constructed in Crystal Ball (Decisioneering, Denver, Colorado), an add-on to Microsoft Excel, which allows a stochastic population growth model to be produced with time steps to match the annual winter estimates of cormorant numbers.

There is much discussion on how to find evidence for density dependence in a time series of population estimates, and how to calculate finite annual growth rates (λ) and determine the variation in this rate when there is natural (environmental) variation and observational error (McCallum 2000; Staples, Taper & Dennis 2004; Freckleton *et al*. 2006). Here, the test of Dennis & Taper (1994) was used, which evaluated the uncertainty in the slope of the density-dependent relationship by using bootstrap Monte Carlo methods (see McCallum 2000). This bootstrapping includes a proportion of iterations where no evidence for density dependence exists (i.e. the slope is ≥ 0). Thus, a proportion, *y*, of iterations use a density-independent relationship, while density-dependent relationships of different strength were used in 1- *y* iterations. To derive a single value for a density dependent growth rate, a slope, intercept and the correlation between them was used to define a predicted growth rate. Estimates of the population size at the start of each year were predicted by:


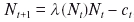
eqn 1

where *N_t_* and *N*_*t*+1_ are the population size in years *t* and *t* + 1 respectively, λ(*N_t_*) is the finite annual growth rate which may be a function of population size in year *t*, and *c_t_* is the number of birds culled during each year. For density independent models, λ(*N_t_*) simplifies to λ. This model makes the pessimistic assumption that culling is additive to natural mortality, although this will be compensated for in the following year within the density-dependent iterations.

### Data

The population data were derived from the Wetland Bird Survey (WeBS) winter cormorant counts for England, from 1986–1987 (when cormorants were first recorded) to 2003–2004. WeBS data are not a population estimate, but are an index of population size with unknown bias. Therefore, although cormorants have been recorded since 1986, the early years may have suffered from under-recording. The British Trust for Ornithology (BTO) recently investigated the validity of data from the early years, and improved the index (Baylis *et al*. 2005). All indices reported here have had missing counts imputed using the Underhill algorithm (Underhill & Prys-Jones 1994) to avoid bias in which sites are recorded in different years. Sites for which data have not been collected on more than 50% of the years are not included in the index. During data validation, there were insufficient counts in 1986–1987 to impute missing values; hence, this year was excluded from all analyses. The above corrections made very little difference to the annual index values. Freckleton *et al*. (2006) report two main sources of observational error that may lead to the spurious detection of density dependence: sampling error and open populations. Given the large physical size of the cormorant (large size is associated with smaller observation error) and the small changes made following data validation, sampling error is judged to be small.

If we assume that a single population supplies the birds available to be counted each winter, then population growth rates between years can be calculated. This single breeding population need not be the English (or British) population; some of the birds that over-winter may migrate from the continent: Wernham *et al*. (1999) estimated that 3·5% of the British wintering population come from outside the UK and Ireland. Unless this varies markedly between years, this will not affect growth rate estimation, as long as there is a degree of over-wintering site fidelity. However, variation in winter immigration from the continent will lead to an increase in the strength of density dependence determined from the data (Freckleton *et al*. 2006). Thus, we cannot be certain that the population is closed, but insufficient data exist to demonstrate closure, or the level of variation. For each year, an estimate of the annual finite growth rate was made, based on the population size in year *t* and *t* + 1. The slope of the density dependent relationship, calculated by the method of Dennis & Taper (1994), will depend upon the population size, rather than just the relative change between years as determined from index values. We refer to the validated BTO data (Baylis *et al*. 2005) as index (1). This index uses counts from all winter months (September to March) and is therefore an index of ‘average bird occupancy’ and is considered the most reliable index (BTO, unpublished data). However, it could be biased if, in some years, birds arrived late and/or left early from the wintering count sites. Therefore, other indices were also used as a comparison. Index (2) used the imputed index values for England prior to the data validation exercise, as used previously in a simple deterministic model of cormorant management (CSL 2004a; 2004b). Index (3) used the maximum winter counts for England. This uses the value from the winter month in which the maximum count was made and is therefore an index of ‘maximum occupancy’. However, this index could be biased as maximum counts could be derived from different months in different years and can be affected by movement between sites (i.e. birds could be double-counted). See Supporting Information Table S1.

Due to bias in the wetland habitat surveyed, it is recognized that WeBS under-records species which are dispersed widely over rivers, non-estuarine coast or small inland waters (e.g. Kershaw & Cranswick 2003; Pollitt *et al*. 2003); this includes the cormorant. A further constraint is that there are gaps in coverage due to some sites not being surveyed on each count date (Kirby 1995; Kershaw & Cranswick 2003). WeBS thus underestimates the true size of the winter cormorant population. However, if this underestimate is approximately stable across years, then index values can be adjusted to give a population estimate.

A number of published sources provide estimates for the magnitude of under-reporting by WeBS (see Supporting Information Table S2). The most recent estimate of the British population is provided by the WeBS Dispersed Waterbirds Survey (DWS), conducted in 2002–2003 (Jackson, Austin & Armitage 2006), which was expected to ‘contribute towards generating reliable wintering waterbird population estimates’ and ‘to provide a new estimate of the numbers of each species wintering in Great Britain’ by adding the extrapolated numbers from the DWS to the 2002–2003 WeBS counts, and is the first to give a confidence interval. The DWS estimate for 2002–2003 is 30 697 (95% confidence limits 20 840 to 46 034). The English cormorant population is estimated at 75% of this value (CSL 2004b). Thus, to estimate the total English cormorant population for each year for each index, the index value for 2002–2003 was assumed to correspond to a population of 23 023 birds (0·75 × 30 697), and the index values were multiplied accordingly.

During 1996/97 to 2002/03 (the latest data available to produce the model), an average of 200 cormorants were shot under licence each year (Table 1). All models constructed therefore assumed that annual growth rates occurred in the presence of 200 birds killed per annum: that is, numbers killed in the model were reduced by 200 in each year to account for this. In 2004/05, the first year in which licences could be issued to kill up to 3000 birds, a total of 1298 were shot (Table 1).

**Table 1:** The number of cormorants licensed to be shot, and the number of cormorants actually shot under licence, for each year since 1996–1997

Year	Licences issued	Birds shot
1996–1997	366	180
1997–1998	443	139
1998–1999	517	167
1999–2000	485	205
2000–2001	506	199
2001–2002	545	225
2002–2003	603	284
Annual mean	495	200
2004–2005	1996*	1298

* 330 licences were issued to kill a maximum of 1996 cormorants.

### Models

Although a model based on index (1) is considered a priori the most ‘correct’ or preferred model, additional models were constructed to evaluate the effect of data interpretation. Four combined models were constructed and each required an estimate for a constant and slope, and the variation around this estimate, sigma, to determine the density dependent finite growth rate. Models (2) and (3) were based on indices (2) and (3) respectively. For models (1) to (3), the Monte Carlo bootstrap method of Dennis & Taper (1994) was used on the least-squares estimates to calculate the uncertainty in each variable, and calculate the *per capita* growth rate, *r*. This was converted to a finite growth rate when used in the models. In each model, a proportion of iterations were run without density dependence, as determined by the proportion of bootstrap iterations that do not support density dependence. In these iterations, a density independent structure was used based on the direct estimate of the annual finite growth rate and its standard deviation. In model (4), process error was ignored, the annual finite growth rate, λ, estimated directly from the annual population estimates:


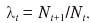
eqn 2

where *N_t_* is the population size in year *t*, and a regression line derived from the data (see Supporting Information Fig. S1), which suggests that density dependence has the formula:


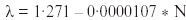
eqn 3

This model differs from the other models in having no uncertainty in the constant or the slope, and this will lead to a much tighter confidence interval around the projected population size. Since it is possible that the strength of density dependence is overestimated (Freckleton *et al*. 2006), a final density independent model (5) was constructed, based on index (1), but where all growth rates were assumed to be density independent.

The initial population size in 2004 was assumed identical to 2003 (i.e. an index value of 100) and 95% confidence limits calculated. Thus the mean starting population for models (1), (4) and (5) was 28 423 (simulated as triangular distributions: minimum 19 296, most likely 28 423, maximum 42 623), for model (2) it was 28 076 (minimum 19 060 and maximum 42 102) and for model (3) it was 24 755 (minimum 16 806, maximum 37 122).

## Results

The density-dependent parameters for all models are given in Table 2, as are the parameters for the growth rate used in those iterations where density dependence was not simulated (i.e. the slope was ≥ 0). This occurred in 0·5% of simulations for model (1), and 0·0% for models (2) and (3), and by definition, in no iterations in model (4) and all iterations in model (5).

**Table 2:** The distributions and values of the instantaneous annual growth rates used by the four models for density-dependent population growth

Model	Distribution	Mean	95th percentile	Mean growth rate (SD)
Model (1)				0.0433
Constant	Normal	0·3679	0·7291	(0·0974)
Slope	Normal	–0·0000158	–0·0000035	
Sigma	Normal	0·0914	0·1287	
Model (2)				0·0573
Constant	Normal	0·4763	0·7592	(0·1147)
Slope	Normal	–0·0000210	–0·0000102	
Sigma	Normal	0·0953	0·1340	
Model (3)				0·0621
Constant	Normal	0·4354	0·6910	(0·1177)
Slope	Normal	–0·0000210	–0·0000101	
Sigma	Normal	0·0974	0·1371	
Model (4)				0·0433
Constant	None	0·2654	NA	(0·0974)
Slope	None	–0·0000107	NA	
Sigma	Normal	0	0·0947	

SD, standard deviation; NA, not applicable

Various levels of annual cull, from 0 to 4000 birds killed per annum were simulated, including the base-line assumption that 200 birds were shot in each year and for each scenario, 10 000 iterations were performed. For the base-line scenario, the percentage of simulations where the population declined by 0 to 50% by 2007, compared to 2004, were calculated (Table 3). If the population was to remain stable at the 2004 value, then we would expect about 50% of simulations to be below a 0% decline. This comparison allowed us to compare the risk of decline between the models. Solving the density-dependent regressions predicted that the population should stabilize at about 23 000 (21 000 birds for model (3) and 25 000 for model (4)), which suggests that the starting population in 2004 is slightly above the carrying capacity. We saw, as expected, a small decline in population size for all density-dependent models (i.e. risk of 0% decline > 50%) and an increase for the density-independent model (i.e. risk of 0% decline < 50%). The mean annual change of the model (1) population under density independence was about +1287 birds, while density dependence suggested a decline of about 1765 birds in the first year and a further 695 birds in the second year.

**Table 3:** The percentage of simulations where the population in 2007 declined by more than a given percentage from the starting population in 2004 for all models; assuming the current level of 200 birds killed under licence per

	Population decline					
	0%	10%	20%	30%	40%	50%
Model (1)	65	57	48	37	25	15
Model (2)	75	65	52	38	24	11
Model (3)	71	60	47	32	18	7
Model (4)	79	57	30	11	1	0
Model (5)	22	8	2	0	0	0

Under the base-line scenario, models (2) and (3) produced very similar risks of decline to each other, but with less variation in population size than model (1). An even greater difference was seen with model (4), which predicted a 79% chance of some level of population decline, but zero risk of a 50% population decline by 2007, and with model (5), which predicted a continuing population increase.

**Fig. 1:**
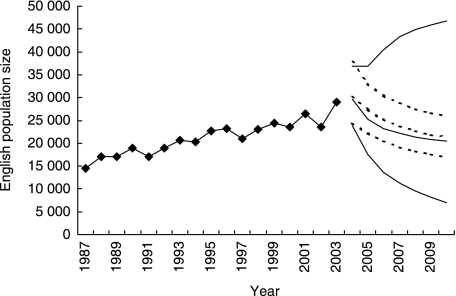
The historical English cormorant population, and the population projection (assuming 1300 birds killed under licence per annum) for two density-dependent models: model (1), solid lines and model (4), dotted lines. For each model, the three lines represent the 90th percentile, the mean and the 10th percentile.

For the scenarios below, the increase in risk (as a percentage of all iterations) through killing birds under licence is presented. If all licensed culling was stopped, then the population would show no real change compared to the base line scenario (Table 4), with the median population being stable rather than decreasing by 2%[Table 5: model (1)]. The risk of a 50% decline by 2007 was 16% greater if 2500 birds were shot each year under licence, and 29% greater if 4000 birds were shot each year [Table 4: model (1)]. If 1300 birds were shot each year, then the risk of a 50% decline was expected to be just 8% greater. This led to a reduction in the median population size of 9%, compared to 2% under the 200-bird scenario [Table 5: model (1)]. Population projection, with the baseline 200 birds shot (see Supporting Information Fig. S2), suggested that the population declined slightly over 6 years. If 1300 birds were shot each year, model (1) predicted that the median population declined to the 1997 level after 6 years, but continued to decline (Fig. 1). We did not simulate the population for longer than this because further annual population estimates would be available with which to refine the model, and change the number of licences granted, as appropriate.

**Table 5:** The median percentage change in the cormorant population (from the 5-year average: 1999–2003) by 2007 for given levels of birds shot each year under licence. Positive values relate to a median population growth above the 5-year average

Licensed birds shot	Model (1) (%)	Model (2) (%)	Model (3) (%)	Model (4) (%)	Model (5) (%)
4000	–32	–33	–40	–30	–10
3500	–27	–30	–32	–25	–5
3000	–23	–26	–29	–20	2
2500	–20	–22	–25	–16	8
2000	–15	–17	–21	–11	14
1500	–10	–13	–17	–7	20
1300	–9	–12	–15	–5	23
1000	–8	–10	–12	–2	27
500	–4	–7	–9	2	33
200	–2	–6	–6	4	37
0	0	–3	–4	6	40

**Table 4:** The percentage increase in risk of a given percentage decline by 2007, for different numbers of birds killed per annum under licence, for model (1). Positive values mean an increase in risk. The ‘Licensed birds shot’ column gives the number of cormorants killed in each year from 2004 to 2006

	Model (1)					
Licensed birds shot	0%	10%	20%	30%	40%	50%
4000	16	20	23	27	29	29
3500	15	17	21	24	25	25
3000	12	14	16	19	20	20
2500	10	12	14	16	17	16
2000	9	10	11	13	15	13
1500	6	6	8	9	9	8
1300	5	6	7	8	8	8
1000	3	4	4	5	6	4
500	1	1	1	1	2	2
0	–1	–1	–2	–1	–1	–1

Models (2) and (3) gave slightly higher risks of decline than model (1) (Table 5). Model (4) suggested a high risk of moderate decline, but a reduced risk of a severe decline, as the number of birds shot each year increased. This was in agreement with the much tighter confidence interval in the population projection with this model (see Figs 1 and S2). Model (5) predicted that with 1300 birds shot per year, the population stabilized near the current level (about 25 000 birds), with a relatively tight confidence interval, and thus limited chance of a significant decline.

A sensitivity analysis of the models was performed by removing the variability or uncertainty of each parameter in turn. This revealed that the risk of population decline was very robust to uncertainty in the variables for measuring the average population size [i.e. a 0% decline: see Table 6 for model (1)]. The risk of a 50% decline by 2007 was robust to uncertainty in the parameters except for the constant and the correlations, with risk changing by up to 7% from the baseline model.

**Table 6:** A sensitivity analysis for model (1) to investigate the effect of uncertainty on each parameter. This shows the percentage of simulations in which the population in 2007 will have declined by a given level (0% or 50%) compared with the population size in 2004. In each year, 1300 birds were killed under licence and each variable in turn was fixed to a single value for all iterations (i.e. uncertainty was removed)

	Per cent simulation with more than	
Variable fixed	0% decline	50% decline
None	71	22
Initial population size	68	19
Sigma	69	21
Slope	73	23
Constant	72	15
Correlations	67	26

## Discussion

There are many different methods used to model population growth and management, and a large variety of approaches to investigate model sensitivity. However, there are only a small number of proposed methods to specifically compare between different model structures. This may be by qualitative comparison (e.g. Smith *et al*. 1995) or more formal information-theoretic approaches (Burnham & Anderson 2002; White & Lubow 2002), although these have been criticized (Stephens *et al*. 2005). Here, rather than choose between different models, we used a single model that included all potential structures, and matched the weight of evidence to support each structure with the proportion of iterations performed for each scenario. This is similar to using Akaike's Information Criteria (AIC) and weighting the output of each model by the delta AIC values (Burnham & Anderson 2002), although this is only done for nested models. In our case, we weighted the models by the percentage of bootstrap iterations that supported each model structure, and noted that results from the full model (1) were in general agreement with models both with and without density dependence. We propose a generalized use of this approach to weight model structure for models with both univariate and multivariate causality (Stephens *et al*. 2005), and note that the models structures do not need to be nested. This could include any aspect of a model (e.g. uncertainty in Allee effects, sex-biased dispersal) for which standard hypothesis testing does not discount a particular structure.

For our example, there has been a clear growth in the number of over-wintering cormorants in England since records began. We utilized population count data in the models and looked for evidence of density dependence at the population level. The use of more detailed biological data (e.g. survival and productivity) in a model would have necessitated a greater number of assumptions about the existence and form of density dependence, and thus, the simpler model was used. The population index suggested that the over-wintering population has doubled since 1987, and that, in the 5 years prior to 2003–2004, the population was relatively stable. The index values were translated into population estimates, with a confidence interval, by using the latest available information (Jackson, Austin & Armitage 2006), and the data were checked for density dependence (Dennis & Taper 1994). In 10 000 bootstrap iterations, 99.5% suggested that density dependence existed; thus, in 99.5% of the simulations, density dependence was incorporated within the model with varying strengths. For those iterations where density dependence did not exist (i.e. the slope of the relationship was ≥ 0), density-independent growth was simulated. Thus, the model encompassed all of the uncertainty associated with the interpretation of the data. The evidence for density dependence is supported by its detection in the general European cormorant population (Frederiksen & Bregnballe 2000; Frederiksen, Lebreton & Bregnballe 2001).

Population projections indicated that the Monte Carlo bootstrap method (Dennis & Taper 1994) produced very wide confidence intervals, due to the uncertainty in the slope of the density-dependent relationship. This variance was so high that the model predicted a substantial risk of a 50% decline over 3 years (15%) with no change in the numbers of licences issued, and predicted that the median population would decline from its current level (29 000). However, sensitivity analysis revealed no major driving parameter for the variance. The model suggested an additional 1000 birds could be culled in 2004–2005 without any real effect on the short-term dynamics of the population, and the current level of culling (1298 birds shot in 2004–2005) would lead to a 5–8% increase in risk of population decline over 3 years. With the previous level of licensed culling (200 birds shot per year), the median population was expected to be 2–4% below the long-term average by 2007, and if the 2004–2005 culling levels were repeated for 3 years, the median population will be reduced by 9%.

The mean growth rate of the inland cormorant population has been between 4% and 6% per annum. Given a current population estimate of 29 000 birds, the licensed shooting in 2004–2005 removed 4·5% of the population, which also suggests that this level of culling is sustainable in the short-term. A longer-term assessment is not necessary as the number of licences permitted in any year can be adjusted annually following the availability of new WeBS data.

Model (1) predicted a small decline from the 1999–2003 average by 2007, if 1300 cormorants were shot each year. After the first year of such a cull, model (1) predicted a decline of 10% below the 2003–2004 high. The WeBS data for 2004–2005 indicated a decline of 6% on the 2003–2004 index (unpublished data). This is consistent with model (1) and model (4), but less consistent with models (2): a 15% decline, (3): a 14% decline, and (5): a 1% increase. In the 2005–2006 winter season, there were 1598 birds shot and the WeBS data indicated a decline of 17% on the 2003–2004 index (unpublished data). Model (1), with 1300 birds shot in 2004 and 1600 birds in 2005 gave an average decline of 15%, thus supporting this model further.

In addition to the imputed and validated index of mean over-wintering bird occupancy (Baylis *et al*. 2005), the unvalidated index and a validated index of maximum bird occupancy were used. These indices had lower values in the early years, resulting in greater evidence for density dependence (100%). The population time series used here is non-stationary and has had a two-step process to reduce observation error (data validation and missing values imputed); thus, the process-error approach of Dennis & Taper (1994), with a Monte Carlo bootstrap method, allowed a weight of evidence approach to density dependence.

The models based on indices (2) and (3) gave similar results. However, when compared to model (1), they gave a different distribution of risk (Table 3), indicating that an incorrect index can give biased results. If we compared the two models based on index (1), model (1) and model (4), we see similar median levels of population decline particularly at high licensing levels (Table 5), but a very different variance (Table 3 and Figs 1 and S2), with model (4) underestimating the risk of population decline. With a much longer time series of data, it is possible that the variance for model (1) will decrease toward that seen for model (4), but analysis of the data does not currently support that degree of accuracy. The larger variance in predicted population size was due to uncertainty in both the slope and the constant used in defining density dependence. Thus, model (4) was not biased, but captures less uncertainty than was actually present. Since the data were not sufficient to predict accurate population projections, the output in Table 4, or the difference between the projections (c.f. Figs 1 and S2) was of greater utility in determining the effects of changes to the number of birds killed under licence. Model (5) assumed complete density independence and was a valid comparison model, since if the detection of density dependence is incorrect, then this model was the most accurate. However, model (5) predicted that a continuing cull of 3000 birds per annum would lead to a relatively stable population (Table 5) and lends further support to the management decision on the level of licensing, since neither this model nor model (1) predicted a significant increased risk of population decline for an annual cull of 1300 birds per year.

The models were only used to project forward 3 years. With stochastic models, uncertainty is compounded over time, making model projections less and less useful for policy makers. Natural resource managers are frequently presented with scenarios where there are uncertainties regarding the effects of policy decisions. Williams *et al*. (2002) describe Adaptive Resource Management (ARM) as a powerful tool for scientific management in these cases. In ARM, emphasis is placed on decision-making to reach a long-term resource goal and defines the information needed to improve management in the future. Thus, information about system responses to management is gathered continuously as decisions are being made, and this information is used to iteratively revise understanding of the system and thus improve decision-making.

A good example of ARM is the annual assessment and setting of North American wildfowl harvest regulations, where decisions are based on resource status and model predictions. The effects are monitored, the information used to refine the original model, and the revised model used for another iteration of the process. The overall system is designed to identify optimal regulatory choices and track model reliability over time (Johnson *et al*. 1997).

Our model suggested that if 200 birds are shot per year, then in 2007 the population would average 28 200 (14 000– 46 000: 80% Certainty Interval), whereas if 1300 birds were shot each year, the population would average 26 300 (12 000–44 000: 80% CI). ARM would involve the annual prediction of the effects of licences to meet an established goal. This would guide the decision to set the number of licences, the effects of this on the population would be monitored through WeBS, and the predicted and observed effects compared. The additional data would be used to improve the model, re-weight the evidence for density dependence and the predictive process would be repeated each year.

The importance of an iterative approach can be illustrated from the outcome of a culling programme conducted on double-crested cormorants in the St Lawrence River Estuary, Québec (Bedard *et al*. 1995). Initial modelling predicted levels of shooting of tree-nesting breeders and egg-oiling to reduce the population over a 5-year period. Monitoring during the programme revealed greater-than-anticipated declines and shooting was stopped after 4 years.

Shooting is known to reduce cormorant activity at inland fisheries (Parrott *et al*. 2003), but we do not know whether a new level of culling will reduce economic damage, or indeed whether there are other equally suitable methods. The annual reassessment of the model allows the risk of more substantial declines to be assessed and managed on an annual basis. The iterative remodelling, using a model which incorporates all structural uncertainty, and assessment inherent in ARM provides a further safeguard to protect the conservation status of the species.

## Supporting Information

Table S1 WeBS cormorant indices

Table S2 WeBS correction values

Fig. S1. Annual cormorant population growth rates.

Fig. S2. Population projection assuming previous licensing rates.
