# Supplementary material for: Managing wildlife populations with uncertainty: cormorants Phalacrocorax carbo
Source: J Appl Ecol. 2008 Dec;45(6):1675–82. doi: 10.1111/j.1365-2664.2008.01380.x (PMC2695860; doi:10.1111/j.1365-2664.2008.01380.x)
Supplement: Supplementary file 1 [file jpe0045-1675-SD1.doc]

**Table S1**. The three WeBS-based population indices for over-wintering cormorants in England. All indices, based on accumulated local sightings, are adjusted to 100 in the winter of 2003/4.

| Year | Validated Index  (bird months)  (1) | Un-validated Index  (2) | Validated Index (maximum count)  (3) |
| --- | --- | --- | --- |
| 1986/87 | N/K | Not used | N/K |
| 1987/88 | 50 | 40 | 37 |
| 1988/89 | 59 | 55 | 53 |
| 1989/90 | 59 | 59 | 59 |
| 1990/91 | 65 | 67 | 69 |
| 1991/92 | 59 | 59 | 58 |
| 1992/93 | 65 | 65 | 63 |
| 1993/94 | 71 | 71 | 70 |
| 1994/95 | 70 | 70 | 67 |
| 1995/96 | 78 | 78 | 79 |
| 1996/97 | 80 | 80 | 75 |
| 1997/98 | 72 | 73 | 73 |
| 1998/99 | 79 | 79 | 79 |
| 1999/00 | 84 | 83 | 85 |
| 2000/01 | 81 | 81 | 91 |
| 2001/02 | 91 | 91 | 89 |
| 2002/03 | 81 | 82 | 93 |
| 2003/04 | 100 | 100 | 100 |
