# Supplementary material for: Managing wildlife populations with uncertainty: cormorants Phalacrocorax carbo
Source: J Appl Ecol. 2008 Dec;45(6):1675–82. doi: 10.1111/j.1365-2664.2008.01380.x (PMC2695860; doi:10.1111/j.1365-2664.2008.01380.x)
Supplement: Supplementary file 2 [file jpe0045-1675-SD2.doc]

**Table S2**. Estimated values for the correction factor to estimate the English population from the WeBS count, and the associated estimate with the year for which the estimate was made. Only the latest value, and its confidence interval, was used in the population models.

| WeBS correction factor | English Population Estimate (2001) | Year factor estimated^††^ | Reference |
| --- | --- | --- | --- |
| 1.24 | 15,338 | 1990-92 | (Kirby, 1995) |
| 1.67 | 20,656 | 1990-94 | (Kershaw & Hughes, 1997) |
| 2.19 | 27,088 | 1997 | (Hughes, Kirby & Rowcliffe, 1999) |
| 1.60 | 19,790 | 1994-98 | (Kershaw & Cranswick, 2003) |
| Not given | 23,023^‡^  (15,630-34,525) | 2002 | (Jackson, Austin & Armitage, 2006) |

^††^The year 1990 refers to the winter of 1990/91, etc.

^‡^The English population and range (95% confidence limit) were calculated as 75% of the GB total, the average percentage for the years 1993-2001.

**References**

Hughes, B., Kirby, J. & Rowcliffe, J.M. (1999) Waterbird conflicts in Britain and Ireland: Ruddy Ducks *Oxyura jamaicensis*, Canada Geese *Branta canadensis*, and Cormorants *Phalacrocorax carbo*. *Wildfowl*, **50**, 77-99.

Jackson, S.F., Austin, G.E. & Armitage, M.J.S. (2006) Surveying waterbirds away from major waterbodies: implications for waterbird population estimates in Britain. *Bird Study*, **53**, 105-11.

Kershaw, M. & Cranswick, P.A. (2003) Numbers of wintering waterbirds in Great Britain, 1994/1995-1998/1999: I. Wildfowl and selected waterbirds. *Biological Conservation*, **111**, 91-104.

Kershaw, M. & Hughes, B. (1997). Trends in the numbers of cormorants *Phalocrocorax carbo*, goosanders *Mergus merganser* and red-breasted mergansers *M. serrator* wintering in the UK. Wildfowl and Wetlands Trust Report to The Britsh Trust for Ornithology, Slimbridge.

Kirby, J.S. (1995) Winter population estimates for selected waterfowl species in Britain. *Biological Conservation*, **73**, 189-98.
