# Supplementary material for: Managing wildlife populations with uncertainty: cormorants Phalacrocorax carbo
Source: J Appl Ecol. 2008 Dec;45(6):1675–82. doi: 10.1111/j.1365-2664.2008.01380.x (PMC2695860; doi:10.1111/j.1365-2664.2008.01380.x)
Supplement: Supplementary file 3 [file jpe0045-1675-SD3.doc]

**Figure S1.** The estimated finite annual growth rate of the English cormorant population, derived by using index (1) from Table S1, plotted against population size. The regression line is shown for the data, and is significant (*P*<0.005). The finite growth rate equals unity at approx. 25,300 birds.
