# Supplementary material for: Managing wildlife populations with uncertainty: cormorants Phalacrocorax carbo
Source: J Appl Ecol. 2008 Dec;45(6):1675–82. doi: 10.1111/j.1365-2664.2008.01380.x (PMC2695860; doi:10.1111/j.1365-2664.2008.01380.x)
Supplement: Supplementary file 4 [file jpe0045-1675-SD4.doc]

**Figure S2**. The historical English cormorant population (solid lines and symbols), and the population projection from 2004 (assuming current licensing levels: 200 per annum) for two density dependent models. Model (1), with solid lines, includes parameter and structural uncertainty, while model (4), dotted lines, has no uncertainty in parameter estimates and is completely density dependent. For each model, the three lines represent the 90^th^ percentile, the mean and the 10^th^ percentile.
